# A Cancer Vaccine Induces Expansion of NY-ESO-1-Specific Regulatory T Cells in Patients with Advanced Melanoma

**DOI:** 10.1371/journal.pone.0048424

**Published:** 2012-10-26

**Authors:** Lisa M. Ebert, Sarah E. MacRaild, Damien Zanker, Ian D. Davis, Jonathan Cebon, Weisan Chen

**Affiliations:** Ludwig Institute for Cancer Research (Melbourne-Austin Branch), Melbourne, Australia; New York University, United States of America

## Abstract

Cancer vaccines are designed to expand tumor antigen-specific T cells with effector function. However, they may also inadvertently expand regulatory T cells (Treg), which could seriously hamper clinical efficacy. To address this possibility, we developed a novel assay to detect antigen-specific Treg based on down-regulation of surface CD3 following TCR engagement, and used this approach to screen for Treg specific to the NY-ESO-1 tumor antigen in melanoma patients treated with the NY-ESO-1/ISCOMATRIX^TM^ cancer vaccine. All patients tested had Treg (CD25^bright^ FoxP3^+^ CD127^neg^) specific for at least one NY-ESO-1 epitope in the blood. Strikingly, comparison with pre-treatment samples revealed that many of these responses were induced or boosted by vaccination. The most frequently detected response was toward the HLA-DP4-restricted NY-ESO-1_157–170_ epitope, which is also recognized by effector T cells. Notably, functional Treg specific for an HLA-DR-restricted epitope within the NY-ESO-1_115–132_ peptide were also identified at high frequency in tumor tissue, suggesting that NY-ESO-1-specific Treg may suppress local anti-tumor immune responses. Together, our data provide compelling evidence for the ability of a cancer vaccine to expand tumor antigen-specific Treg in the setting of advanced cancer, a finding which should be given serious consideration in the design of future cancer vaccine clinical trials.

## Introduction

Cancer vaccines hold great promise in the treatment of solid tumors such as melanoma, and have been the focus of extensive pre-clinical and clinical testing in recent years. Due to its exceptional immunogenicity, NY-ESO-1 has emerged as one of the most promising targets in such approaches [1]. Over the last several years, we have conducted a series of clinical trials in melanoma patients using a cancer vaccine consisting of full-length recombinant NY-ESO-1 protein formulated with ISCOMATRIX^TM^ adjuvant (CSL Limited, Australia). Although this vaccine had potent anti-tumor effects in pre-clinical animal studies [2] and showed promising results in the initial Phase I study [3], it failed to significantly improve clinical outcome in melanoma patients in subsequent trials [4] and manuscript in preparation). Furthermore, while patients with fully resected (early-stage) disease developed strong effector T cell (Teff) responses to NY-ESO-1 following vaccination [3], [5], patients with advanced melanoma had much less robust responses [4].

Similar to our experience with the NY-ESO-1/ISCOMATRIX^TM^ vaccine, many other cancer vaccines have also failed to induce significant clinical benefit, often despite the induction of seemingly potent tumor antigen-specific Teff responses [6], [7]. There are many potential explanations for this, but one that has received particular attention in recent years centers around the role of CD4^+^ CD25^+^ FoxP3^+^ regulatory T cells (Treg). Treg are essential for preventing autoimmunity [8]. However, a growing body of evidence supports the concept that Treg can also block the generation of effective anti-tumor immunity [9]. It is therefore imperative that cancer vaccine approaches avoid expanding these cells.

Until recently, evidence for the recognition of tumor antigens by Treg had been scarce, and it was unclear whether or not Treg would be activated and expand in response to vaccination against tumor antigens. In recent years, however, a number of reports have identified Treg specific for a range of tumor antigens in human cancer, including NY-ESO-1, survivin, TRP-1, gp100, MAGE-A3, Melan-A, carcinoembryonic Ag (CEA), telomerase, HER2/neu, WT-1, MUC-1 and papillomavirus antigens E6 and E7 [10]–[16]. The presence of these cells in cancer patients raises serious concerns about the potential of cancer vaccines to expand not only Teff but also Treg. The extent to which this actually occurs, however, is poorly understood.

In the present study, we have evaluated the effect of vaccination with NY-ESO-1/ISCOMATRIX^TM^ on the frequency of NY-ESO-1-specific Treg in patients with late-stage melanoma. As most Treg do not produce cytokines upon activation [17]–[19], there is currently no suitable assay available to screen for antigen-specific Treg. We have therefore developed a novel, systematic approach in which antigen-specific Treg are detected by down-regulation of surface T cell receptor (TCR)/CD3 complexes following *in vitro* stimulation with a library of short antigenic peptides. The optimization of this method has recently been described [20]. Here, we have used this approach to screen for NY-ESO-1-specific Treg in melanoma patients, before and after vaccination with the NY-ESO-1/ISCOMATRIX^TM^ vaccine. This study has enabled us to gain an unprecedented understanding of tumor antigen-specific Treg in the setting of advanced cancer, including their function, location, the range of epitopes recognized and how their frequency is affected by vaccination.

## Results

### A novel approach based on down-regulation of surface CD3 detects Teff and Treg specific for NY-ESO-1 peptides

In order to screen for NY-ESO-1-specific Treg in an unbiased manner, we developed an assay based on the principle that T cells down-modulate the number of CD3/TCR complexes on the cell surface following the binding of cognate antigen [21], [22]. This can be detected as a reduced level of cell surface CD3 staining by flow cytometry. Due to their scarcity, NY-ESO-1-specific Teff can almost never be detected directly *ex vivo* in blood samples using standard (IFN-γ intracellular cytokine staining) methodology but instead require prior expansion *in vitro*. Preliminary studies revealed that this was also the case for NY-ESO-1-specific Treg detected using the CD3 down-regulation method (unpublished observations). Accordingly, patient PBMC were cultured with a panel of 28 partially overlapping 18 amino acid peptides which collectively span the entire sequence of NY-ESO-1 [23], to allow expansion of NY-ESO-1-specific Teff and Treg to detectable frequencies. Conditions, including culture time, IL-2 concentration and source of serum, were optimized for Treg expansion [20]. After 21d expansion, cultures were re-stimulated with individual 18mer peptides and the level of surface CD3 determined by flow cytometry, in conjunction with staining for Treg markers.

**Figure 1:**
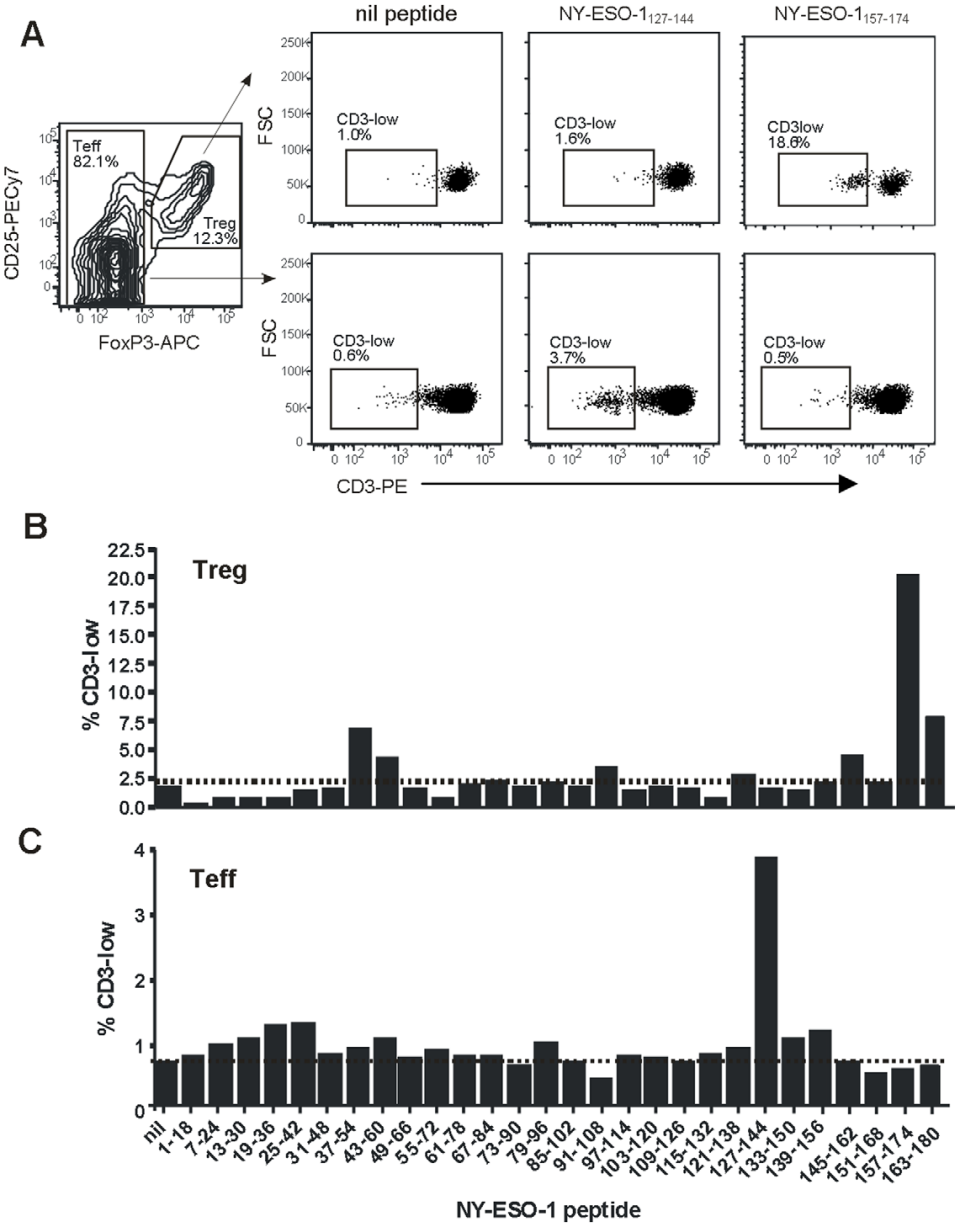
Antigen-specific Treg and Teff can be detected by measuring down-regulation of surface. *CD3*. PBMC from Patient 113 were cultured with pooled NY-ESO-1 18mer peptides as described in *Methods*, followed by re-stimulation with the indicated individual peptides during overnight culture to allow CD3 down-regulation. Cells were stained with antibodies to CD4, CD25, FoxP3 and CD3 and analyzed by flow cytometry. (***A***): an example of the staining patterns observed, illustrating the gating of Treg and Teff (on gated viable CD4^+^ T cells) and the down-regulation of CD3 within each population. (***B***–***C***): A summary of the responses detected within the Treg (B) and Teff (C) populations. Dotted lines indicate the baseline level of CD3-low cells (nil peptide condition).

An example of the results obtained is shown in Figure 1. These data demonstrate that a clear population of putative Treg could be detected within the CD4^+^ T cell population by co-staining for CD25 and FoxP3 (Fig 1A, left). Within this Treg subset, a distinct sub-population of CD3-low (antigen-specific) cells was apparent after re-stimulation with peptide NY-ESO-1_157–174_ but not NY-ESO-1_127–144_ or in the absence of re-stimulation (Fig 1A, upper panels). Conversely, a CD3-low sub-population could be detected within the Teff subset following re-stimulation with peptide NY-ESO-1_127–144_ but not NY-ESO-1_157–174_ (Fig 1A, lower panels). A summary of results for this patient using all 28 peptides demonstrates two distinct Treg responses, within regions NY-ESO-1_37–60_ and NY-ESO-1_157–180_ (Fig 1B), and one major Teff response, within the region NY-ESO-1_127–144_ (Fig 1C).

We also sought to analyze the cytokine production profile of NY-ESO-1-specific Treg in four patients whose Treg down-regulated CD3 in response to NY-ESO-1 peptides, using intracellular cytokine staining. CD3-low Treg produced low to undetectable levels of IL-4, IL-10 and IL-17 in all patients. However, production of IFN-γ and TNF-α was variable, with two patients showing negligible production by the CD3-low Treg, and two patients showing high levels of production (Figure S1). The secretion of pro-inflammatory cytokines such as IFN-γ by Treg has been described previously [16], [24]–[26], and appears to be a unique feature of Treg identified in cancer patients. However, our results show that this characteristic is sporadic, and can't be relied on for Treg identification.

### Cells identified by the CD25^+^ FoxP3^+^ phenotype have phenotypic and functional characteristics of Treg

**Figure 2:**
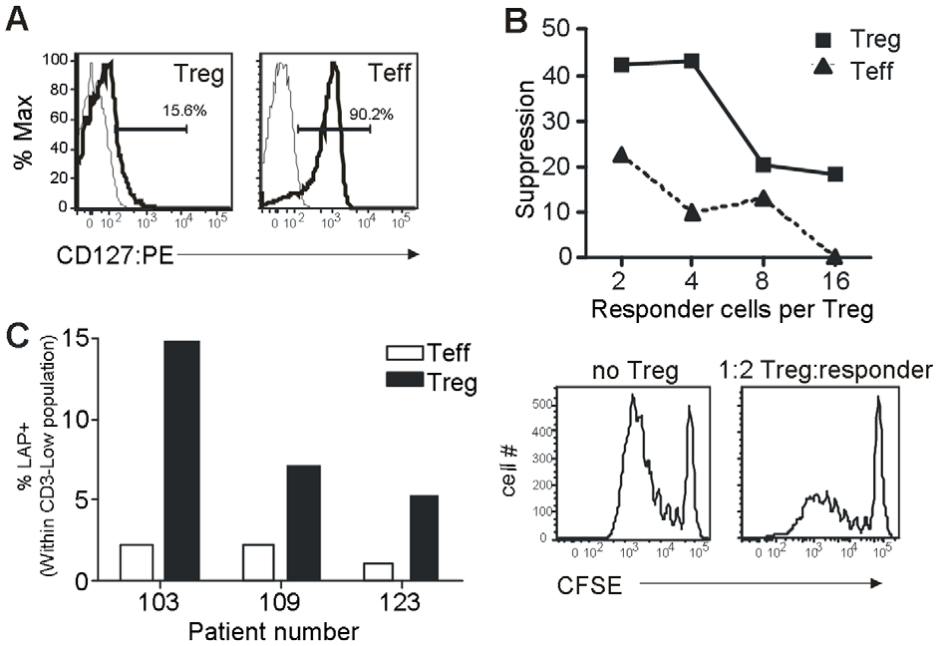
CD4^+^ CD25^+^ FoxP3^+^ cells identified after culture have phenotypic and functional characteristics of Treg. Patient PBMC were cultured for 21d with NY-ESO-1 18mer peptide(s) known from preliminary studies to induce a Treg response and then: (***A***) analyzed for CD127 expression by flow cytometry, gating on Treg (CD4^+^ CD25^+^ FoxP3^+^) or Teff (CD4^+^ FoxP3^−^) as indicated. Results shown are representative of three experiments with similar results; or (***B***): Treg were purified by sorting CD4^+^ CD25^+^ CD127^low^ cells and tested for their ability to suppress the proliferation of CFSE-labeled CD8^+^ T cells pre-stimulated for 16hr with plate-bound anti-CD3. The graph shows % suppression relative to cultures conducted in the absence of Tregs, while flow cytometry histograms below illustrate CFSE profiles obtained for responder T cells alone (left) or at 1∶2 ratio with Treg (right). Data are representative of three independent experiments using samples from three different individuals. In (**C**), cultured PBMC were re-stimulated with the relevant peptide and expression of LAP on the surface of Tregs or Teffs was determined by flow cytometry within the CD3-low (peptide-responsive) population for three patients.

Both CD25 and FoxP3 are known to be transiently induced on Teff after activation, meaning that activated Teff can potentially be mistaken for Treg [27]–[30]. Given the extended culture period used (21d), it is unlikely that the cells identified as CD4^+^ CD25^+^ FoxP3^+^ in our culture system represent activated Teff, since expression of FoxP3, and to a lesser extent CD25, returns to baseline after ∼10 days [12], [27], [28], [30]. However, to further demonstrate that these cells are indeed Treg, cultures were co-stained with an antibody to CD127, which is expressed at greatly reduced levels on Treg compared to Teff [31], [32]. Figure 2A demonstrates that cells identified as Treg (CD4^+^ CD25^+^ FoxP3^+^) expressed barely detectable levels of CD127, whereas cells identified as Teff (CD4^+^ FoxP3^−^) expressed uniformly high levels. Furthermore, when bulk Treg were FACS-sorted from 21d cultures (according to a CD4^+^ CD25^+^ CD127^−/low^ phenotype), these cells induced a dose-dependent decrease in the proliferation of CD8^+^ T cells (Fig 2B).

Finally, we sought to determine whether antigen-specific activation of Treg led to induced expression of the TGF-β latency-associated peptide (LAP) on the cell surface. Induction of LAP expression following activation is a characteristic unique to Treg, and this molecule is not expressed on Teff cells, even after activation [18], [33]. In order to directly compare activation-induced LAP expression on Treg and Teff, we identified three patients in which the Treg and Teff populations both down-regulated CD3 in response to the same peptide. PBMC from these patients were cultured for 21d with peptide, re-stimulated overnight and assessed for CD3 down-regulation and surface LAP expression. As shown in Figure 2C, LAP was clearly induced on a sub-population of Treg responding to NY-ESO-1 peptide, as identified by the CD3-low phenotype. In contrast, CD3-low Teff responding to the same peptide failed to up-regulate LAP.

Together, the expression of a CD4^+^ CD25^+^ FoxP3^+^ CD127^−^ phenotype coupled with *in vitro* suppressive capacity and the ability to induce LAP expression strongly suggest that the cells analyzed here are functional Treg rather than activated Teff which have temporarily up-regulated FoxP3 expression.

### Down-regulation of CD3 by Treg is dependent on peptide concentration

**Figure 3:**
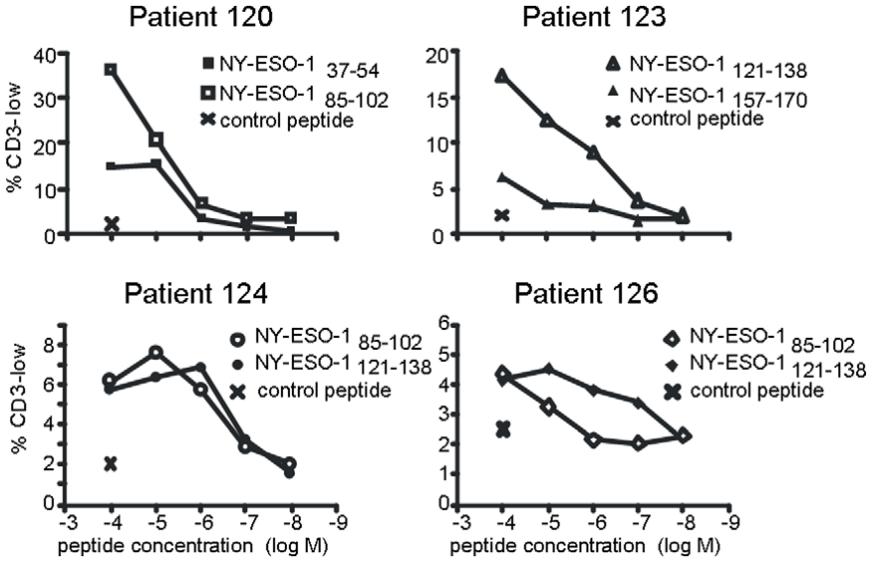
CD3 down-regulation on Tregs is dependent on the concentration of peptide used for re-stimulation. PBMC from four patients were cultured for 21d with NY-ESO-1 18mer peptides and then re-stimulated overnight with individual peptides at the indicated concentrations. The proportion of Treg down-regulating CD3 in response to peptide was determined by flow cytometry.

In this study, down-regulation of surface CD3 is used as a marker of antigen-specific activation of Tregs via the TCR. Thus, the extent of CD3 down-regulation would be expected to be strictly dependent on peptide concentration. To confirm this, peptide titration studies were performed using PBMC from 4 different patients. For each patient, two peptides were tested, which were selected on the basis of preliminary screening experiments as being able to induce a CD3 down-regulation response at 1×10^−4^M. As shown in Figure 3, there was a clear dose-dependent relationship between the proportion of CD3-low Treg and the concentration of peptide used for re-stimulation. A number of these responses could be titrated down to 1×10^−7^M peptide, which is comparable with several previously described CD4^+^ Teff responses to NY-ESO-1 peptides [5], [34]. It is also important to note that the 18mer peptides used here likely do not constitute the optimal epitope for T cell recognition, in which case the response detected when peptide concentration becomes limiting may be a considerable underestimate.

### NY-ESO-1-specific Treg are frequently detected in the blood of late-stage melanoma patients and can be expanded by the NY-ESO-1/ISCOMATRIX^TM^ vaccine

**Figure 4:**
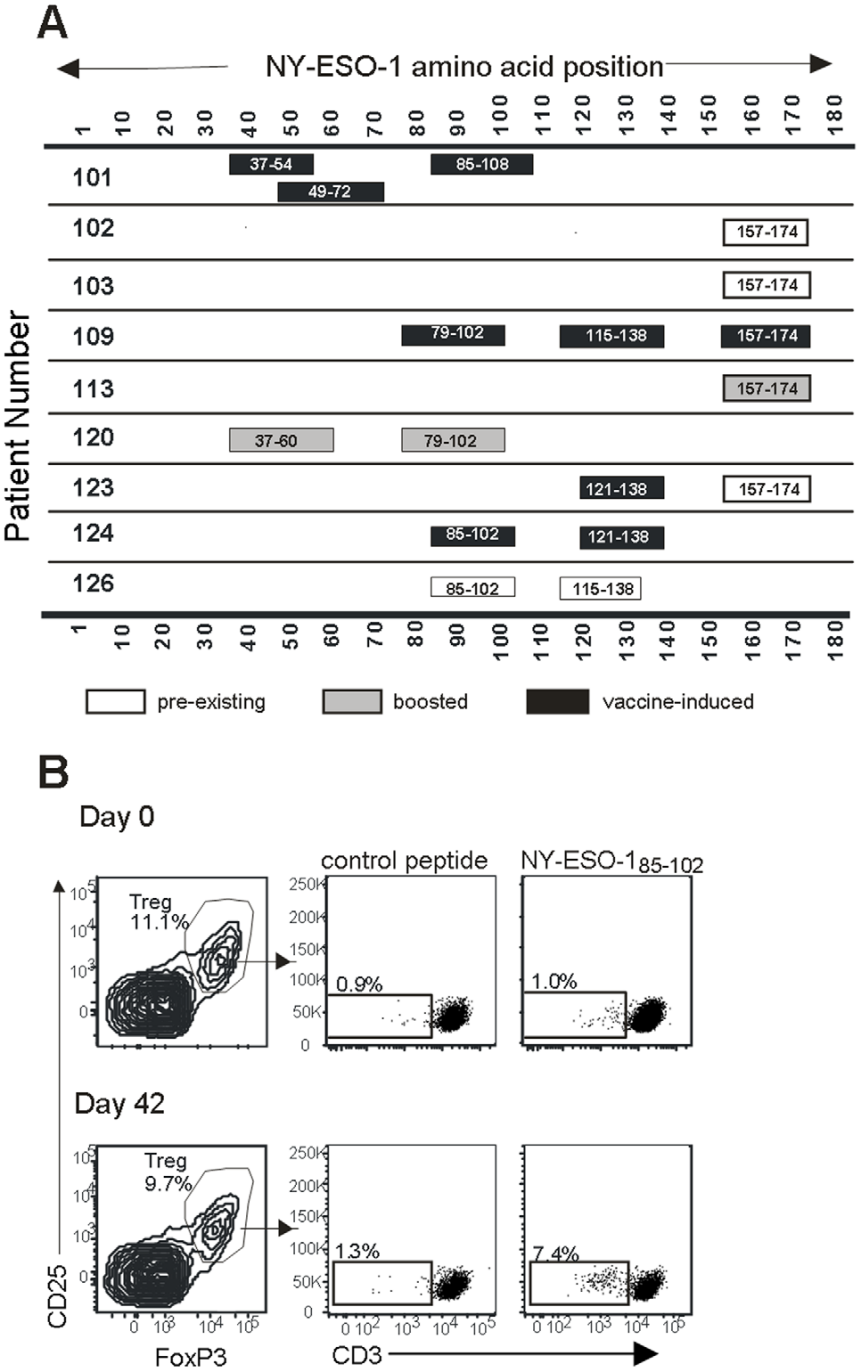
Summary of NY-ESO-1-specific Treg responses detected in a cohort of 9 patients vaccinated with NY-ESO-1/ISCOMATRIX^TM^ vaccine. (***A***): For every patient within the cohort, each validated Treg response is summarized with a box. Responses are considered validated if they were observed in at least two independent cultures, using two independently synthesized batches of peptide. The position of the box indicates where in the NY-ESO-1 peptide sequence the response was localized. In the event that responses were detected to two peptides adjacent in sequence, this is shown as a single response spanning the two peptides. Shaded boxes indicate that the magnitude of the response was increased at least 2-fold in post-vaccination samples compared to pre-vaccination samples when both samples were tested in parallel under identical conditions. Solid boxes indicate that the response was only detectable in samples collected after vaccination. Open boxes indicate that the magnitude of the response was similar pre- and post-vaccination. (***B***): An example of a response that was induced by vaccination (Patient 124) is shown. Treg were gated on the basis of CD25 and FoxP3 expression, and CD3 down-regulation was assessed following re-stimulation with either control peptide or the same peptide used for expansion (NY-ESO-1_85–102_).

A cohort of nine patients with advanced melanoma was screened for NY-ESO-1-specific Treg, both before and 42 days after vaccination with NY-ESO-1/ISCOMATRIX^TM^. The results are summarized in Figure 4A. After vaccination, all patients had Treg responses specific to at least one region of the NY-ESO-1 protein; several had responses to two or three distinct regions. Strikingly, a comparison with pre-vaccination samples revealed that many of these responses were induced by the vaccine, as they were undetectable prior to vaccination. An example of such a response is shown in Figure 4B. In addition, some responses that were detectable prior to vaccination appeared to be boosted by the vaccine (defined as a >2-fold increase in responsive cells after vaccination, when the two samples were compared in parallel under identical conditions). For example, in Patient 120, the frequency of Treg specific to peptide NY-ESO-1_37–54_ was 4.4% before vaccination but 18.3% after vaccination, and the frequency of Treg specific to peptide NY-ESO-1_79–96_ was 3.3% prior to vaccination and 16.3% after vaccination.

### Treg and Teff respond to an identical epitope in the region NY-ESO-1_157–170_

**Figure 5:**
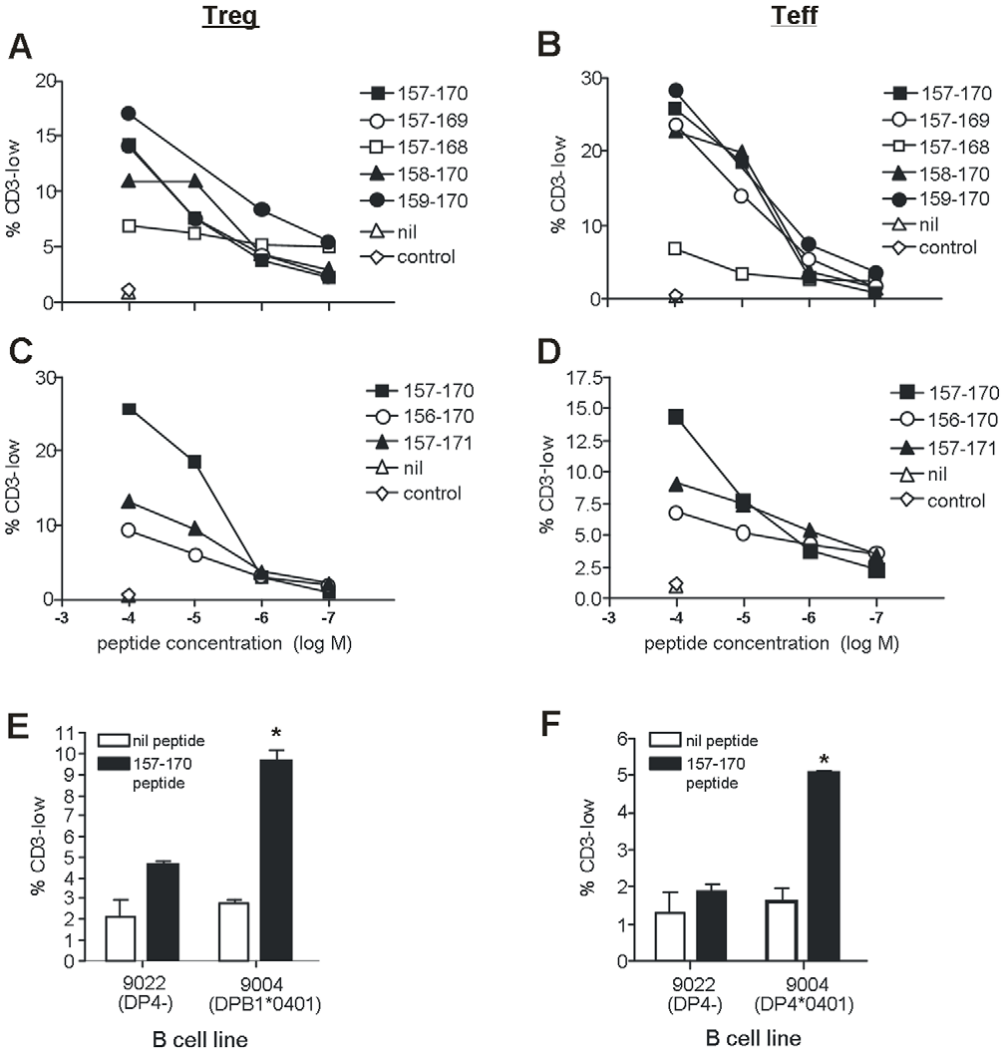
Treg and Teff respond to an identical HLA-DP4-restricted epitope in the region NY-ESO-1_157–170_. Patient PBMC were cultured for 21d with the 18mer peptide NY-ESO-1_157–174_ and then re-stimulated with the indicated short HPLC-purified peptides by either adding directly to the culture as usual (***A–D***) or by pulsing onto BCL followed by washing (***E–F***). After overnight incubation, cells were stained and analyzed by flow cytometry, gating on Treg (CD4^+^ CD25^+^ FoxP3^+^; ***A, C and E***) or Teff (CD4^+^ FoxP3^−^; ***B, D and F***). Peptides used for re-stimulation were based on the published epitope NY-ESO-1_157–170_, with either truncation (***A–B***) or extension (***C–D***) at each terminus. Graphs in ***A–D*** show results obtained for Patient 102; similar results were also obtained for Patient 103. Graphs in ***E–F*** show mean + SEM from Patients 102 and 113; an asterisk indicates a p value of <0.05 (t test).

Figure 4A demonstrates that Treg responses were most commonly seen toward the peptide NY-ESO-1_157–174_, with Tregs specific to this 18mer peptide detectable in 5/9 patients tested. This region contains an epitope (NY-ESO-1_157–170_) that has previously been characterized for Teff [35], raising the possibility that Treg could be responding to the same epitope. To address this possibility, two patients (102 and 103) were identified who had responses to the NY-ESO-1_157–174_ peptide in both the Treg and Teff subsets, and the ability of these cells to respond to the published epitope (NY-ESO-1_157–170_) or various truncation or extension variants, was assessed. As shown in Figure 5, both Treg (left panels) and Teff (right panels) responded to the NY-ESO-1_157–170_ peptide. Truncation of either 1 or 2 aa from the N-terminus, or 1 aa from the C-terminus, had little effect on these responses. However, both populations showed greatly reduced responsiveness following truncation of 2 aa from the C-terminus (peptide NY-ESO-1_157–168_). Similarly, extension of the core sequence by 1 aa at either the C or N terminus also reduced responsiveness within both populations.

The previously described NY-ESO-1_157–170_ epitope has been shown to be restricted by the HLA-DP4 class II allele [35], and molecular typing revealed that the patients tested in Figure 5 expressed the HLA-DPB1*0401 molecule. To confirm that Tregs also responded to this epitope when it was presented on HLA-DP4, a panel of EBV-transformed B cell lines (BCL) was pulsed with the NY-ESO-1_157–170_ peptide, washed and tested for the ability to induce CD3 down-regulation in Treg and Teff cells (Fig 5E–F). A BCL lacking DP4 expression (9902) failed to induce CD3 down-regulation in either population, whereas a BCL expressing HLA-DPB1*0401 (line 9004) induced a significant response in both populations. Thus, the NY-ESO-1_157–170_ peptide is presented to both Treg and Teff on HLA-DP4.

Together, these results suggest that the minimum sequence of the previously described NY-ESO-1_157–170_ epitope [35] could be revised to NY-ESO-1_159–170_, or possibly even to NY-ESO-1_159–169_, although this exact peptide was not tested in our assays. More importantly, however, the data demonstrate that Treg and Teff respond to exactly the same epitope, and in both cases, this response is restricted by HLA-DP4.

### NY-ESO-1-specific Treg are prevalent within tumor tissue

To determine if NY-ESO-1-specific Treg can be detected within the tumor as well as the blood, fresh tumor tissue was obtained from Patient 126 after three rounds of vaccination with NY-ESO-1/ISCOMATRIX^TM^ and a single cell suspension generated. The resulting mixture of tumor cells and stromal cells, including TIL, was cultured in the presence of high-dose IL-2 but without the addition of any exogenous peptide, antigen or mitogen. Expanded TIL were stimulated overnight with peptides NY-ESO-1_85–102_ and NY-ESO-1_115–132_ (both of which induced responses in peripheral blood Treg from this patient) and assessed for CD3 down-regulation. In addition, a sample of the tumor digest was analyzed by flow cytometry prior to culture, which revealed that 41.1% of CD4^+^ T cells within the tumor had a Treg phenotype, representing an approximate 10-fold enrichment over frequencies in the blood (not shown).

**Figure 6:**
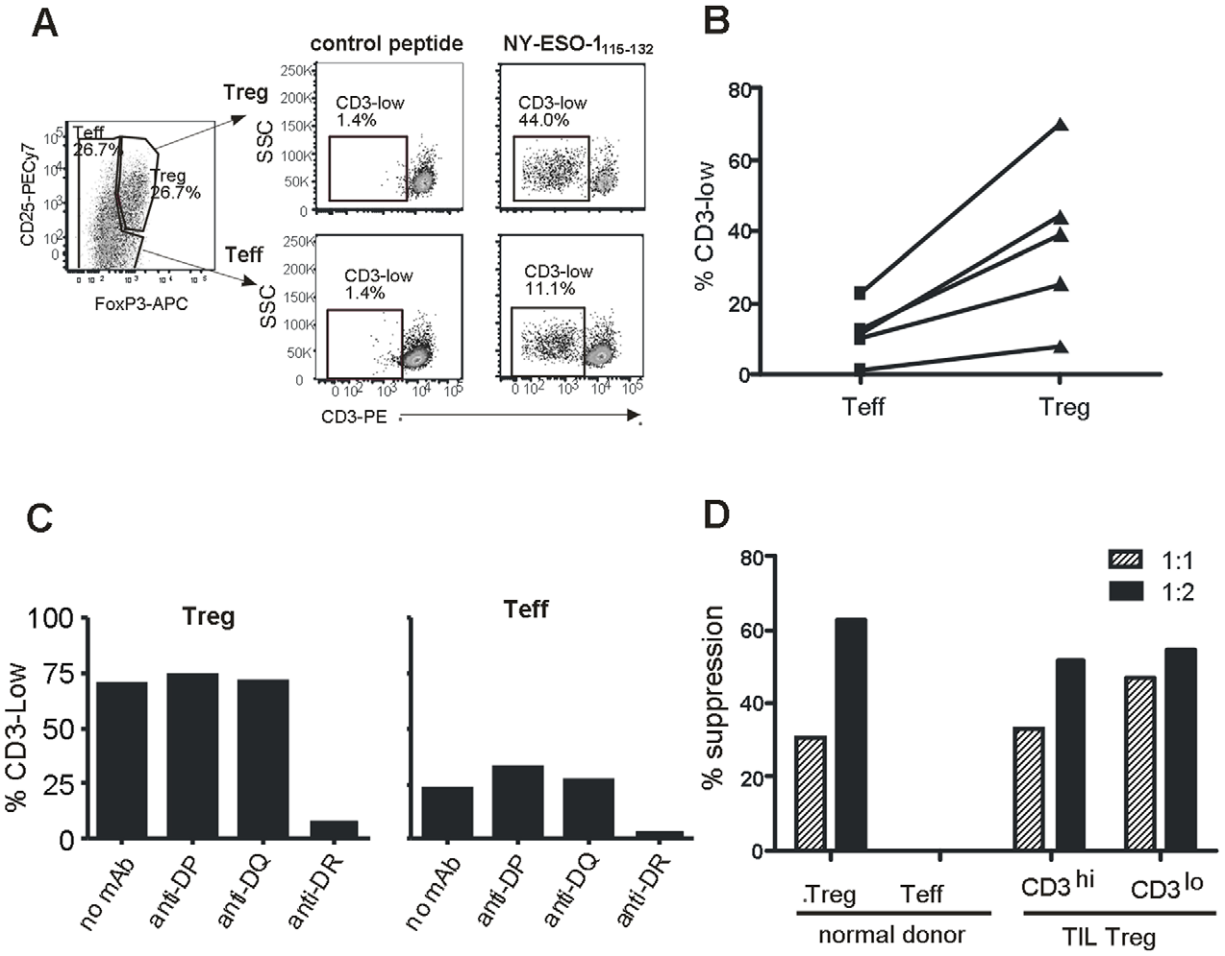
Tumor tissue contains populations of NY-ESO-1-specific Treg and Teff. Tumour tissue was obtained from Patient 126 and TIL lines generated as described in *Methods*. (***A–B***): Cells were treated overnight with peptide NY-ESO-1_115–132_ or control peptide, and then stained and analyzed by flow cytometry, gating on Treg (CD4^+^ CD25^+^ FoxP3^+^) or Teff (CD4^+^ FoxP3^−^) as indicated. Representative flow cytometry dot plots (**A**) show the gating of Treg and Teff, and the CD3 down-regulation response observed in each population following re-stimulation, while (**B**) shows a summary of results obtained in five experiments, each using one of the three different TIL lines generated. (***C***): The effect of blocking antibodies to HLA-DR, HLA-DP or HLA-DQ on the response to peptide NY-ESO-1_115–132_ within Treg (left) and Teff (right) populations. Similar results were obtained in a second experiment. (***C***): TIL were stimulated overnight with NY-ESO-1_115–132_ peptide and then peptide specific (CD3^lo^) and non-specific (CD3^hi^) Treg (CD4^+^ CD127^lo^ CD25^hi^) were purified by cell sorting and tested for their ability to suppress the proliferation of CFSE-labeled CD8^+^ T cells pre-stimulated for 4hr with anti-CD3 at the indicated Treg:responder ratios. As a comparison, Treg were also sorted from previously frozen PBMC obtained from a healthy donor. Similar results were observed in a second experiment, although higher Treg:responder ratios were required to see suppression.

Peptide NY-ESO-1_85–102_ failed to stimulate any reproducible responses by either Treg or Teff within TIL. On the other hand, peptide NY-ESO-1_115–132_ induced a readily detectable response by Teff cells within the expanded TIL (Fig 6A). Strikingly, the magnitude of response to the same peptide was ∼4-fold higher within the Treg population, with >40% of Treg down-regulating CD3 in response to this peptide. To demonstrate the reproducibility of this difference, three independent TIL lines were generated from frozen aliquots of the same tumor specimen, and each line tested 1–3 times. In each analysis, the proportion of cells responding to the NY-ESO-1_115–132_ peptide was always higher within the Treg population than the Teff population, with a mean fold difference of 3.1 (Fig 6B). Pre-incubation with a blocking antibody to HLA-DR almost completely inhibited both the Treg and Teff responses to this peptide, whereas blocking antibodies to HLA-DP or HLA-DQ had no effect, indicating that the response within both populations was restricted by HLA-DR (Fig 6C).

In Figure 2, we demonstrated that bulk Treg isolated from 21-day cultures suppressed the proliferation of CD8^+^ T cells. However, it was also of interest to confirm that NY-ESO-1-specific Treg had a similar activity. Expanded TIL from Patient 126 were stimulated with NY-ESO-1_115–132_ peptide overnight to induce CD3 down-regulation and the peptide-specific Treg were subsequently sorted on the basis of a CD4^+^ CD25^+^ CD127^−^ CD3^low^ phenotype and tested in a suppression assay. These cells suppressed proliferation of anti-CD3-stimulated CD8^+^ T cells from a healthy donor to a similar extent as bulk Treg isolated from a healthy donor (Fig 6D). Interestingly, CD3^hi^ Tregs sorted from the TIL line (i.e., Treg which had failed to respond to peptide stimulation) also suppressed CD8^+^ T cell proliferation, suggesting that recent antigen stimulation was not essential for the acquisition of suppressor function by these cells. Possibly, the presence of tumor cells during establishment of the TIL line activated Tregs specific to a wide variety of tumor antigenic epitopes, and this activated state was maintained during expansion, thus enabling Treg with a variety of antigen specificities to exert suppressive activity.

Together, these results demonstrate that tumor tissue from Patient 126 contains a prominent population of Treg specific for an HLA-DR-restricted epitope within the NY-ESO-1_115–132_ peptide. Moreover, Treg specific for this epitope can suppress CD8^+^ T cell proliferation and are therefore fully functional. Immunohistochemistry staining of the tumour tissue revealed that tumour cells still expressed high levels of NY-ESO-1 at this time (data not shown), suggesting that tissue-resident NY-ESO-1-specific Treg may be activated locally.

## Discussion

In the present study, we have utilized a novel approach that allows the unbiased identification of Treg specific to any epitope within a given tumor antigen. We demonstrate that NY-ESO-1-specific Treg are very common in the blood of patients with late-stage melanoma, as they were detectable in 9/9 patients tested. Moreover, in six of these nine patients, the NY-ESO-1/ISCOMATRIX^TM^ vaccine either induced at least one new response that was not detectable prior to vaccination or boosted the pre-existing responses.

Due to the scarcity of tumour antigen-specific T cells (including Treg) in the blood, it was necessary to expand these cells in vitro with antigen. This culture step may have induced a temporary up-regulation of FoxP3 and CD25 on Teff cells, as has been reported previously [27]–[30], and these cells could theoretically have been mistaken for Treg. However, several lines of evidence strongly suggest that this is not the case. First, these cells suppressed the proliferation of CD8^+^ T cells, when assessed as either a bulk population (Fig 2) or isolated according to NY-ESO-1 specificity (Fig 6). Second, they expressed low to undetectable levels of CD127 expression. Third, they up-regulated LAP upon activation with cognate peptide. Finally, the Treg and Teff populations in individual patients sometimes recognized distinct epitopes (such as in the example shown in Figure 1), providing evidence that these are discrete populations, and the Treg were not simply generated by conversion from Teff recognizing the same epitope. Our conclusion that CD4^+^ CD25^+^ FoxP3^+^ cells present in 21-day cultures are Treg and not activated Teff is in agreement with previous studies showing that the acquisition of these markers by Teff is temporary, and expression is largely lost by day 10 of culture [12], [27], [28], [30]. Of note, two previous studies have also identified NY-ESO-1-specific Treg in the blood of melanoma patients using alternative approaches, further supporting our findings [12], [24].

The Treg responses detected in our study span a large portion of the NY-ESO-1 protein. Although detailed characterization of each of these epitopes is beyond the scope of this study, we could confirm that the frequently detected Treg response to the 18mer peptide NY-ESO-1_157–174_ was due to recognition of the previously described DP4-restricted immunodominant NY-ESO-1_157–170_ epitope. Thus, we have shown conclusively that Treg and Teff can recognize the exact same minimum epitope presented on the same HLA allele. This in turn suggests that human Treg and Teff share at least partially overlapping TCR repertoires, which is in agreement with previous studies in humans [36] and mice [37]. On the other hand, one of the Treg responses detected here (within the region NY-ESO-1_49–72_) does not contain any previously described Teff epitopes (see http://www.cancerimmunity.org/peptidedatabase) and this response was not detected within the Teff population in this patient (data not shown). Thus, this specificity may be unique to the Treg population.

It is still a matter of debate whether Treg mediate their suppressive effects primarily in the secondary lymphoid organs, at local sites in the periphery (such as tumor tissue) or, more likely, a combination of both. For practical reasons, we have assessed NY-ESO-1-specific Treg responses predominantly in the blood, which should reflect the cells circulating through secondary lymphoid organs. However, for Patient 126, we could additionally obtain fresh tumor tissue and demonstrate that Treg specific for an HLA-DR-restricted epitope within peptide NY-ESO-1_115–132_ were not only present within the blood but were also present at high frequency within the tumor. These NY-ESO-1_115–132_-specific Treg were fully functional, as they suppressed CD8^+^ T cell proliferation. Considering that Patient 126 expresses the HLA-DRB1*04 allele, this response is likely to be directed toward the previously described DR4-restricted NY-ESO-1_121–130_ epitope [34]. The frequency of these Treg was remarkably high, suggesting that local anti-tumour immunity would be severely compromised in this patient.

The potential of cancer vaccines to expand not only desirable Teff populations, but also inhibitory Treg, has important implications for cancer immunotherapy. Cancer patients may be particularly predisposed to developing Treg responses, since the proportion of Treg in the blood is significantly increased above normal for a wide range of cancers [38], [39]. Furthermore, we [4] and others [40] have shown that patients with advanced melanoma have a significantly higher frequency of Treg in the blood compared to those with fully resected disease. In the present study, we have demonstrated that the NY-ESO-1/ISCOMATRIX^TM^ vaccine expands NY-ESO-1-specific Treg in the majority of patients with advanced melanoma. However, it is quite possible that patients with fully resected disease, who have a lower proportion of circulating Treg [4], would be less likely to develop NY-ESO-1-specific Treg responses following vaccination. We are currently testing this hypothesis.

In keeping with our findings, two other clinical studies have shown that tumour antigen-specific Treg can be expanded by cancer vaccines targeting either MAGE-A3 [10] or papillomavirus [14]. In contrast to these reports, two further studies failed to detect an expansion of Treg in response to vaccination [11], [41]. It is likely that the effects of cancer vaccines on tumor antigen-specific Treg vary depending on the vaccinating antigen and the nature and stage of the cancer. Further studies using systematic screening approaches such as that used here are required to fully understand the effect of these and other variables.

Together, our results demonstrate that Treg specific for a range of NY-ESO-1 epitopes are present in both the blood and tumor tissue of patients with advanced melanoma and that vaccination against NY-ESO-1 frequently expands these cells. Thus, it may be important to modify future cancer vaccine approaches, for example, by co-administration of sub-therapeutic doses of the chemotherapeutic drug cyclophosphamide, which has been shown to result in selective depletion of Treg [42]. We are currently assessing whether this approach can deplete Treg and improve responses to the NY-ESO-1/ISCOMATRIX^TM^ vaccine in patients with advanced melanoma. In addition, our results highlight the importance of assessing the effect of vaccination on Treg and not just Teff, in order to fully understand the immune responses (both effector and regulatory) elicited by vaccination.

## Materials and Methods

### Patients and vaccine

The study population and vaccine are described in detail elsewhere [4]. Briefly, all patients had histologically confirmed stage IV (metastatic) or unresectable stage III malignant melanoma. All studies were approved by the Human Research Ethics Committees of Austin Health and the Peter MacCallum Cancer Centre and patients provided written informed consent.

### Peptides, antibodies and dyes for flow cytometry

Peptides were synthesized by the Department of Chemistry, Auckland University, New Zealand) and Chiron Mimotopes. Antibodies for flow cytometry specific to CD3, CD4, CD8, CD25 and CD127 were from BD Biosciences. Anti-LAP was from R&D Systems while anti-FoxP3 (clone 236A/E7) was from eBioscience. Carboxyfluorescein succinimidyl ester (CFSE) and Live/Dead vital dyes for flow cytometry were from Invitrogen. Anti-CD3 (clone OKT3) used for T cell stimulation was from eBioscience. Pan anti-HLA-DR (L243), anti-HLA-DP (B7/21), and anti-HLA-DQ (SPV-L3) neutralizing antibodies were used as culture supernatants [43].

### Flow cytometry and cell sorting

Flow cytometry was performed using a BD FACSCanto II and data was analyzed using FlowJo. ‘Live/Dead’ vital dye was used according to the manufacturer's recommendations to gate out dead cells from analyses. Cell sorting was performed using a BD FACSAria III Cell Sorter.

### Preparation and culture of cell lines, peripheral blood mononuclear cells (PBMC) and tumor-infiltrating lymphocytes (TIL)

Culture medium used throughout was RPMI-1640 supplemented with 2 mM Glutamax, 100 U/ml penicillin, 100 μg/ml streptomycin, 10 mM HEPES, 1mM sodium pyruvate, 100 μM non-essential amino acids and 50 μM 2-Merceptoethanol (Invitrogen). For culture of PBMC and TIL, human serum pooled from several healthy donors (Australian Red Cross) was added to 10% to generate complete ‘RH-10’ medium. Alternatively, fetal calf serum (FCS; Invitrogen) was added to 10% to generate complete ‘RF-10’ medium for other cell lines.

PBMC were isolated from blood by Ficoll-Paque density gradient centrifugation (Amersham) and cryopreserved in 10% dimethyl sulfoxide (DMSO) until required. Healthy donor PBMCs were obtained from the Australian Red Cross. To generate TIL lines, fresh tumor tissue was rinsed to remove blood cells and then dissociated by mincing and digestion in Collagenase III (1 mg/ml) and DNase I (50 U/ml) at 37°C for 1.5 hr, then cultured in RH-10 supplemented with 300 U/ml IL-2 (‘Proleukin’; Prometheus). EBV-transformed B cell lines (BCL) 9022, 9004 and 9072 were made available from the 10th International HLA Workshop (New York).

### Detection of antigen-specific responses using CD3 down-regulation

The method is described in detail elsewhere [20]. Briefly, PBMC were incubated with a pool of 28 18mer peptides for 1 hour at 37°C. Cultures were then diluted ∼10-fold with RH-10 containing 300 U/mL IL-2 and incubated at 37°C for 21 days, and then re-stimulated overnight (16 – 24 hr) with peptide (at 10^−4^M unless otherwise indicated), followed by staining for CD3, CD4, CD25 and FoxP3 using the FoxP3 buffer set from eBioscience. In some experiments, instead of adding peptide directly to the culture, BCL were loaded with 10^−6^M peptide for 60 minutes at 37°C and then washed. The peptide-pulsed BCL were mixed with an equal number of cultured PBMC, incubated overnight and stained for flow cytometry as usual. To determine broad HLA restriction, hybridoma supernatant containing neutralizing anti-HLA antibodies was added to PBMC cultures at a 1/10 dilution and incubated at RT for 30 minutes prior to addition of peptide.

### Suppression Assay

Treg were sorted on the basis of a CD3^+^ CD4^+^ CD25^+^ CD127^−^ phenotype. For some experiments (Fig 6), Treg were stimulated overnight prior to sorting with 1×10^−6^M peptide NY-ESO-1_115–132_ and additionally sorted on the basis of a CD3^hi^ or CD3^lo^ phenotype. Sorted Treg were mixed at the indicated ratios with healthy donor derived CD8^+^ T cells labeled with CFSE and pre-stimulated with plate bound anti-CD3 (1–5μg/ml). Assays were cultured for 4 days before assessing the proportion of divided CD8^+^ T cells, as determined by CFSE dye dilution. In initial experiments (Fig 2), peptide-pulsed BCL generated from the same patient were also added as a source of APC. Suppression was calculated according to the formula: 100×(100-[% divided ‘test’/% divided ‘responders only’]), as described [44].

## Supporting Information

Figure S1 (TIFF)
